# Human mesenchymal stem cells/multipotent stromal cells consume accumulated autophagosomes early in differentiation

**DOI:** 10.1186/scrt530

**Published:** 2014-12-17

**Authors:** Austin Nuschke, Melanie Rodrigues, Donna B Stolz, Charleen T Chu, Linda Griffith, Alan Wells

**Affiliations:** Department of Pathology, University of Pittsburgh, 200 Lothrop St, Pittsburgh, PA 15261 USA; McGowan Institute for Regenerative Medicine, 450 Technology Dr Suite 300, Pittsburgh, PA 15219 USA; Department of Cell Biology, University of Pittsburgh, 3500 Terrace St, Pittsburgh, PA 15261 USA; Department of Biological Engineering, Massachusetts Institute of Technology, 77 Massachusetts Ave, Cambridge, MA 02139 USA; S713 Scaife Hall, 3550 Terrace Street, Pittsburgh, PA 15261 USA

## Abstract

**Introduction:**

Bone marrow mesenchymal stem cells/multipotent stromal cells (MSCs) are recruited to sites of injury and subsequently support regeneration through differentiation or paracrine activity. During periods of stress such as wound site implant or differentiation, MSCs are subjected to a variety of stressors that might activate pathways to improve cell survival and generate energy. In this study, we monitored MSC autophagy in response to the process of differentiation.

**Methods:**

MSC autophagosome structures were observed by using transmission electron microscopy and a tandem green fluorescent protein-red fluorescent protein autophagic flux reporter to monitor the mammalian microtubule-associated protein-1 light chain 3 (LC3) turnover in real time. MSCs were differentiated by using standard osteogenic and adipogenic media, and autophagy was examined during short-term and long-term differentiation via immunoblots for LC3I and II. Autophagy was modulated during differentiation by using rapamycin and bafilomycin treatments to disrupt the autophagosome balance during the early stages of the differentiation process, and differentiation was monitored in the long term by using Von Kossa and Oil Red O staining as well as quantitative polymerase chain reaction analysis of typical differentiation markers.

**Results:**

We found that undifferentiated MSCs showed an accumulation of a large number of undegraded autophagic vacuoles, with little autophagic turnover. Stimulation of autophagy with rapamycin led to rapid degradation of these autophagosomes and greatly increased rough endoplasmic reticulum size. Upon induction of osteogenic differentiation, MSC expression of LC3II, a common autophagosome marker, was lost within 12 hours, consistent with increased turnover. However, during adipogenic differentiation, drug treatment to alter the autophagosome balance during early differentiation led to changes in differentiation efficiency, with inhibited adipocyte formation following rapamycin treatment and accelerated fat accumulation following autophagosome blockade by bafilomycin.

**Conclusions:**

Our findings suggest that MSCs exist in a state of arrested autophagy with high autophagosome accumulation and are poised to rapidly undergo autophagic degradation. This phenotype is highly sensitive, and a balance of autophagy appears to be key in efficient MSC differentiation and function, as evidenced by our results implicating autophagic flux in early osteogenesis and adipogenesis.

## Introduction

Mesenchymal stem cells/multipotent stromal cells (MSCs) have the ability to migrate into sites of injury, self-renew, and differentiate as well as release trophic and growth factors [1–4]. These activities combine to bring about post-injury tissue regeneration, making them prime candidates for use in regenerative medicine, including repair of tissues such as bone and cartilage. For purposes of therapy, MSCs are often implanted into wound beds devoid of nutrients and oxygen and high in reactive oxygen species and pro-inflammatory/pro-death cytokines, which lead to a rapid loss of these cells [5–8]. However, endogenous MSCs contribute to wound healing, despite being subject to the harsh wound microenvironment, suggesting that MSCs have an innate mechanism of adapting to an environment low in nutrients. In other situations, MSCs also face highly demanding conditions during the process of expansion and differentiation, where the cells are used to generate new tissue; this has been studied in the contexts of myocardial repair, epidermal skin healing, and many others [9–12]. In either case, cellular mechanisms that can help the cells prime themselves to efficiently overcome these high metabolic demands would be advantageous to the cell on an innate level and also as potential mechanisms to improve clinical outcomes.

Macroautophagy, a conserved form of autophagy (and called simply autophagy hereafter), is a catabolic process of ‘self-eating’ or cannibalism wherein starving cells fuel themselves by forming double membranous vacuoles called ‘autophagosomes’ that sequester and degrade cytoplasmic material upon fusion with lysosomes. Traditionally, autophagy has been considered a means of recycling cellular components during times of nutrient starvation, and indeed autophagosome formation is prevalent in cells under nutrient deprivation and hypoxia [13, 14]. Additionally, autophagy plays a role in cellular differentiation such as mitochondrial clearance during erythrocyte differentiation or fat droplet deposition during adipocyte differentiation [15]. Previous studies have found autophagosomes to be present in MSCs [16–18] at a level higher than many differentiated cells. This suggested that the autophagosomes are altered during differentiation.

In this study, we queried whether autophagosomes play a role during MSC differentiation and function and thus could be potentially modulated to affect the differentiation process. We used transmission electron microscopy (TEM) and the autophagosome marker LC3II to determine that autophagosomes were more prevalent in the MSCs than the differentiated cells, with the cells being filled with autophagosomes. Using a tandem fluorescent reporter to examine autophagic flux, we found that in MSCs under normal conditions *in vitro* these autophagosomes had not fused with lysosomes and therefore were not being degraded or recycled. Additionally, a forced release from this hold on autophagy led to rapid loss of autophagosomes accompanied by expansion of the rough endoplasmic reticulum (RER) indicative of cellular reprogramming. We further studied differentiating MSCs, showing activation of autophagy during early differentiation and altered differentiation outcomes when autophagy was modulated during the same time period. Our results suggest a mechanism by which MSCs are arrested in mid-autophagy while being maintained as multipotent cells, allowing rapid generation of autophagosome degradation products when needed. This function could allow the cells to adapt to challenging conditions in wound healing and ultimately may be a hallmark of a stem cell.

## Materials and methods

### Reagents

Dulbecco’s modified Eagle’s medium (DMEM) (10-014-CV) was obtained from CellGro (Manassas, VA, USA). RNase Minikit (74104) and Quantitect Reverse Transcription Kit (205311) were obtained from Qiagen (Valencia, CA, USA), and Brilliant SYBR Green Quantitative Polymerase Chain Reaction (qPCR) Master Mix (600548) was from Agilent Technologies (Santa Clara, CA, USA). Bafilomycin A1 from *Streptomyces griseus* (B1793) was obtained from Sigma-Aldrich (St. Louis, MO, USA) and tamoxifen citrate (54965-24-1) from ICN Biomed (Irvine, CA, USA).

For immunoblotting, rabbit polyclonal LC3 antibody (NB100-2331) was obtained from Novus Biologicals (Littleton, CO, USA) and goat anti-rabbit IgG secondary antibody (A9169) was from Sigma-Aldrich. Housekeeping gene anti-β-actin produced in rabbit (A2668) was from Sigma-Aldrich. The protein ladder for all immunoblots was a Full Range Rainbow marker (RPN800E) from GE Life Sciences (Pittsburgh, PA, USA).

### Cell culture

Primary human bone marrow-derived multipotential stromal cells (prhMSCs) were obtained from Darwin Prockop at the National Institutes of Health-funded Stem Cell Repository at Texas A&M University. These cells were deemed exempt by the University of Pittsburgh Institutional Review Board as they are provided without any protected health information or identifiers and do not require the University of Pittsburgh to obtain informed consent. These procedures comply with the Declaration of Helsinki. Cells were maintained in alpha minimum essential medium (α-MEM) without ribonucleotides or deoxyribonucleotides, supplemented with 16.5% fetal bovine serum (FBS) (Atlanta Biologicals, Lawrenceville, GA, USA), 100 units per mL penicillin/streptomycin and 2 mM L-glutamine. Cells between P1 and P3 were used for experiments. A second human bone marrow-derived cell line, immortalized by using human telomerase reverse transcriptase (ihMSCs), a kind gift from Junya Toguchida’s lab, Kyoto University, Japan, was also used for studies. Proliferation media comprising DMEM with 10% FBS (Gemini Bio-Products, West Sacramento, CA, USA), containing 1 mM sodium pyruvate, 1 mM L-glutamine, 1 μM non-essential amino acids, and 100 units per mL penicillin-streptomycin, was used.

To account for changes in oxygen concentration, cells were grown in either incubators at ambient air conditions (21% oxygen) or Biospherix incubators (Biospherix, Lacona, NY, USA) at 4% oxygen. At no point in the culture of cells at 4% oxygen were the cells exposed to ambient oxygen conditions, as the microscopes were also contained in the Biospherix chamber.

For osteogenic differentiation media, proliferation media was supplanted with 100 nM dexamethasone, 100 μM β-glycerophosphate, and 50 μM L-ascorbic acid. Cells were maintained in osteogenic differentiation media at either 21% or 4% oxygen for up to 30 days. For adipogenic differentiation, the hMSC adipogenic bulletkit (PT-3004) from Lonza (Hopkinton, MA, USA) was used throughout differentiation, and induction and maintenance media were switched every three days.

### Transmission electron microscopy

MSCs were grown to confluence in a six-well plate in 5.5 mM glucose and left untreated or treated with 5 μM rapamycin or 5 nM bafilomycin for 1 hour or 4 hours. After treatment, cells were fixed in 4% formaldehyde, washed in phosphate-buffered saline (PBS), and processed as previously described [19]. Sections (70 nm) were imaged on a Jeol JEM-1101 transmission electron microscope (Jeol, Peabody, MA, USA), and autophagosomes were captured at different magnifications as described in the results.

### Osteogenic differentiation

Immortalized human MSCs (ihMSCs) (up to passage 15) were incubated in osteogenic DMEM for 72 hours (short term) or 30 days (long term) at 21% or 4% oxygen. In short-term studies, protein lysates were collected after 1, 3, 6, 12, 24, 48, and 72 hours of differentiation. Conditions were included to modulate autophagy by using 5 μM rapamycin or 5 nM bafilomycin for the first 3 hours of differentiation to control short-term autophagy outcomes. Protein lysates for all differentiations were analyzed for changes in LC3 via immunoblot. LC3II bands were normalized to β-actin control bands for each time point and then standardized via sum of replicates for each individual blot across three experiments [20]. Average normalized LC3II band density was calculated with the resulting values for each experimental condition. For long-term studies, samples were collected after 0, 10, 20, and 30 days, with hydroxyapatite deposition probed by Von Kossa stain. Briefly, samples were washed with PBS without calcium or magnesium, fixed in 4% paraformaldehyde, treated with 1% silver nitrate solution, and incubated under ultraviolet light for 10 minutes. Wells were washed with sodium thiosulfate overnight and imaged under transmitted light.

RNA was isolated from MSCs grown in osteogenic media on days 0, 10, 20, and 30 by using an RNeasy kit (Qiagen). After treatment with genomic DNA wipeout buffer, 1 μg template RNA was reverse-transcribed into cDNA by using a Quantitect cDNA kit (Qiagen). qPCR was performed by using 1 μL cDNA for all samples with glyceraldehyde 3-phosphate dehydrogenase (GAPDH) as control, 12.5 μL of Brilliant SYBYR green master mix (Agilent Technologies), and 3.75 μL forward and reverse primers. Primers for Runx2 and Osteocalcin, markers for early and late osteogenesis, were used to probe for osteogenesis. Forward and reverse primer sequences were the following:

- GAPDH forward primer: 5′-GAGTCAACGGATTTGGTCGT-3′
- GAPDH reverse primer: 5′-TTCATTTTGGAGGGATCTCG-3′
- Runx2 forward primer 5′-CCTCGGAGAGGTACCAGATG-3′
- Runx2 reverse primer 5′-TTCCCGAGGTCCATCTACTG-3′
- Osteocalcin forward primer 5′-GTTTATTTGGGAGCAGCTGGGATG-3′
- Osteocalcin reverse primer 5′-GTTTATTTGGGAGCAGCTGGGATG-3′.

### Adipogenic differentiation

Up to passage 10, ihMSCs were grown to confluence in standard DMEM (see above) at 21% oxygen. Samples were then treated with adipogenic differentiation media (Lonza, see above) and assayed for fat droplet formation after 10, 20, and 30 days. To monitor the effects of modulating early autophagy on the long-term differentiation process, separate groups were treated with 5 μM rapamycin or 5 nM bafilomycin for the first 3 hours of differentiation, after which normal adipogenic media was used for the rest of the 30-day study.

At each time point, fat droplet accumulation was assayed by using Oil Red O staining. For the Oil Red O reagent, stock solutions were prepared by using 3 mg/mL Oil Red O (O-0625) (Sigma-Aldrich) in isopropanol. Working solutions consisted of 3:2 Oil Red O stock in deionized water. For staining, cells were fixed in 4% paraformaldehyde, washed, and treated with 60% ethanol for 5 minutes. Oil Red O stain was added for 5 minutes, and samples were imaged for fat droplet formation, with droplets appearing red under transmitted light. Adipocyte differentiation was interpreted as the percentage area occupied by fat droplets. This was calculated by using ImageJ by converting all 20× images to 8-bit format and reducing threshold to highlight the red adipocytes in culture, using the same thresholding for all analyzed images. Particle analysis in ImageJ was set to detect any droplets with a radius greater than 75 pixels to exclude debris and miniscule droplets. Percentage areas of each image occupied by adipocytes were averaged by using eight fields across two experiments, and percentage area measurements were compared at days 20 and 30 of differentiation when significant fat accumulation was seen.

### Assessment of autophagosome turnover

Lipofectamine 2000 (Invitrogen, Carlsbad, CA, USA) was used to transfect MSCs with a monomeric red fluorescent protein-green fluorescent protein (mRFP-GFP) tandem fluorescently tagged LC3 plasmid (tfLC3), a kind gift from Tamotsu Yoshimori’s lab, Osaka University, Japan [21]. The plasmid is designed in a manner that both RFP and GFP are expressed in autophagosomes on LC3 until fusion with the lysosome. Once in the acidic compartments of the lysosome, GFP-LC3 fails to fluoresce while RFP-LC3 continues to generate a fluorescent signal. For transfection, 1 μg of plasmid was diluted in Optimem and mixed in a 1:1 ratio with Lipofectamine 2000 diluted 1:25 in Optimem. This mixture was incubated for 20 minutes at room temperature and added to MSCs grown to 70% confluence in 5.5 mM or 25 mM glucose media without FBS. MSCs were allowed to sit in this mix for 6 hours, after which cells were placed in complete media. Media without FBS was used instead of Optimem because of MSC death in the presence of Optimem alone.

Cells were left untreated, to probe for basal autophagosome recycling, or treated with 5 μM rapamycin (LC Laboratories, Woburn, MA, USA) or 5 nM bafilomycin (B1793-2UG) (Sigma-Aldrich) for 15 minutes or 1 hour and imaged for RFP and GFP fluorescence. Whereas yellow (merge of GFP and RFP) puncta indicate autophagosomes prior to fusion with lysosomes, red puncta indicate autophagosomes post fusion with lysosomes [21].

### Statistical analysis

In analyses for osteogenic marker expression and fat droplet percentage area, all treatments were compared by using a Student’s *t* test, and a *P* value of less than 0.05 was considered statistically significant.

## Results

### Mesenchymal stem cells contain a large number of mid-stage autophagosomes

**Figure 1:**
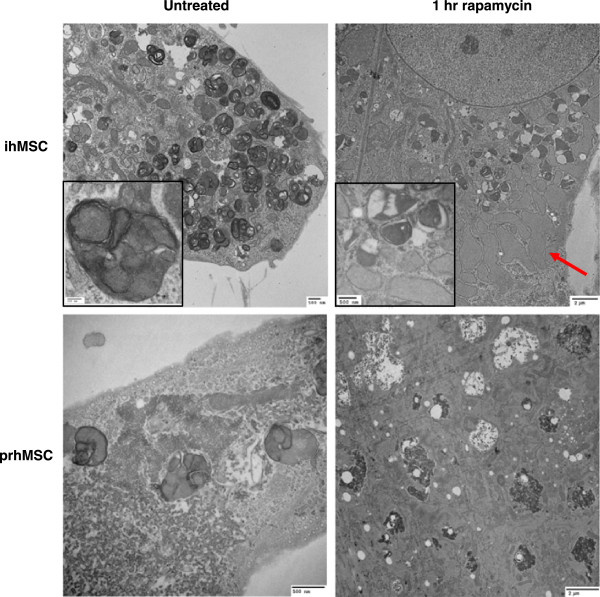
**Mesenchymal stem cells (MSCs) show a high number of autophagosomes.** Immortalized human MSCs grown at standard culture conditions (1 g/L glucose, 10% fetal bovine serum, or FBS) or following treatment with 5 μM rapamycin for 1 hour were subjected to transmission electron microscopy analysis at 15,000× magnification (top). A 150,000× image of a late-stage autolysosome is included with the control treatment (top left inset), as is a 40,000× magnification of residual autophagosome structures in the rapamycin treatment (top right inset). Primary human MSCs were analyzed for autophagosome formation at standard conditions (1 g/L glucose, 16.5% FBS) at 40,000× (bottom left) and following 5 μM rapamycin treatment for 1 hour at 10,000× (bottom right). ihMSC, immortalized human mesenchymal stem cell; prhMSC, primary human mesenchymal stem cell.

We have earlier reported that MSCs generate reactive oxygen species despite being largely glycolytic [5, 22, 23]. As such, we examined the mitochondrial content of ihMSCs by using TEM. Interestingly, we found a large number of mitochondria, but unexpectedly these were at least partially encapsulated in autophagosomes (Figure 1). The autophagosomes do not appear to have fused with lysosomes, and the ratio of early autophagosomes to late/‘empty’ ones is abnormally high (Figure 1) and thus they seem to have accumulated in a partial arrest prior to lysosomal degradation. In contrast, 1-hour treatment with rapamycin, a drug that inhibits mammalian target of rapamycin (mTOR) signaling and stimulates autophagy [24], led to rapid clearance of previous autophagosome clusters that also coincided with an increase in RER size (Figure 1). After 4 hours of rapamycin treatment, the remaining autophagosomes seem to have cleared completely, and the RER size returned to a more normal state (data not shown). Early stages of new autophagosome formation appear to emerge after 4 hours of rapamycin stimulation, consistent with a new cycle of autophagosome generation. This pattern of autophagosome utilization was not present in untreated MSCs.

Analysis of prhMSCs shows a similar phenotype though not as striking as what is seen with the telomerase-immortalized cells (Figure 1). Notably, the smaller cells in prhMSC cultures were the ones that showed the higher autophagosome concentrations, in contrast with a more constant high concentration in the immortalized cells. Treatment with rapamycin for 1 hour led to autophagosome degradation as seen in the ihMSCs; RER enlargement was also noted though again to a lesser extent than in the immortalized MSCs (Figure 1). Nevertheless, the autophagosome accumulation and changes upon induction were qualitatively similar in both the immortalized and primary human MSCs.

### Autophagy is highly active during the early stages of mesenchymal stem cell differentiation

**Figure 2:**
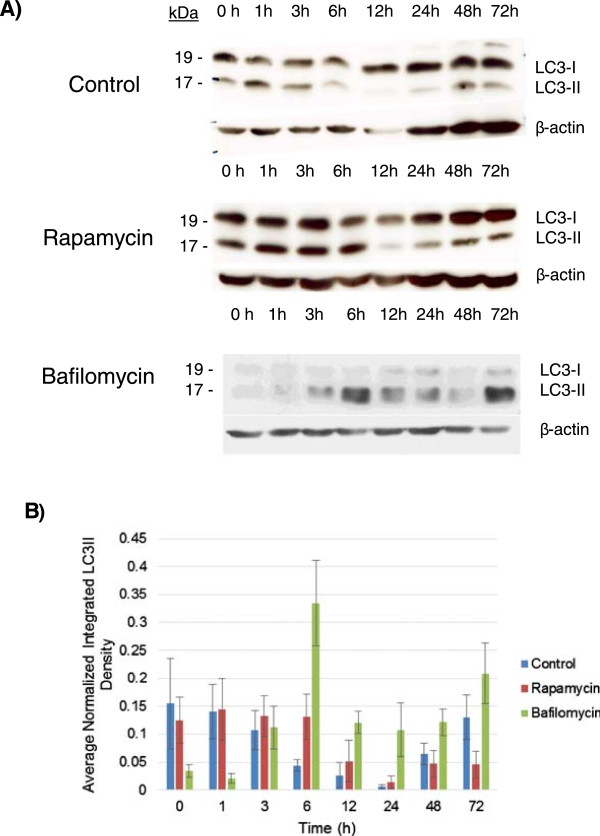
**High autophagosome concentration is consumed during early immortalized human mesenchymal stem cell differentiation. (A)** Immortalized human mesenchymal stem cells were differentiated under osteogenic conditions (see Materials and methods) and assayed for changes in LC3I and LC3II during a 72-hour window. Cells were differentiated under standard conditions (top) or with addition of 5 μM rapamycin (middle) or 5 nM bafilomycin (bottom) for the first 3 hours of differentiation to modulate autophagy. Immunoblots were performed for LC3 at the indicated time points to assess autophagosome degradation via relative changes in LC3II (lower band; 17 kDa). Studies were repeated three times with similar trends seen consistently. **(B)** Average standardized densities normalized by the sum of replicates were quantified via densitometry to measure autophagosome accumulation (LC3II bands) across three separate differentiations. Average values as standardized to β-actin are reported here. LC3, light chain 3.

Recent evidence has suggested that autophagy occurs in MSCs and more importantly plays a role in a critical early period (approximately 3 hours) during the differentiation process [18]. We first looked at how MSCs responded to the first few days of differentiation with respect to changes in LC3, a common marker for autophagosome degradation. Immortalized MSCs were grown to confluence and exposed to standard osteogenic differentiation media for 72 hours, and lysates were collected for immunoblot analysis of LC3 at various time points (Figure 2A). To examine changes in LC3 compared with standard drug treatments to modulate autophagy, we also included treatments of rapamycin and bafilomycin (a drug that inhibits autophagy) in separate groups to control MSC autophagy during the aforementioned critical window, followed by replacement of the media with the normal differentiation media (Figure 2A).

About 15 autophagy-related proteins act in a hierarchical manner to bring about initiation, nucleation, elongation, and recycling of the autophagosomes [25]. The mammalian microtubule-associated protein-1 light chain 3 (LC3) is part of the elongation-closure machinery of autophagosomes. It is present either in a form unconjugated to lipids called LC3I that is widely distributed in the cytoplasm or in a form conjugated to the lipid phosphotidylethanolamine and bound to both membranes of the mature autophagosome called LC3II [26]. LC3II is a commonly used marker for autophagosomes since it migrates to a lower position during electrophoresis [27]. LC3 immunoblotting is generally assessed by the relative changes in LC3II (lower band) over time, signifying the degradation of autophagosomes by the lysosome, which decreases LC3II that has been localized to the autophagosome membrane. Osteogenic differentiation results in Figure 2A and B show a marked and rapid reduction LC3II, decreasing from a high initial level across the course of treatment, with a notable reduction in LC3II just after 3 hours of differentiation stimulus (with relatively constant cytosolic LC3I, as seen in the blots in Figure 2A and quantitated by densitometry across repeat experiments in Figure 2B). There was also an apparent recovery following the initial activation of autophagic flux, as cytosolic LC3I levels increased at 24 hours and a subsequent partial recovery of LC3II levels was observed (Figure 2B).

This control differentiation showed a trend similar to the rapamycin-treated cells, which showed a similar rapid clearance of LC3II and subsequent re-accumulation of LC3 after 24 hours of differentiation. This effect was inhibited by bafilomycin treatment, which limited LC3II turnover and generally inhibited LC3II degradation, most notably at the 6-hour mark when the average LC3II density was greatly increased in the bafilomycin treatment compared with the control and rapamycin treatments. Bafilomycin treatment also led to a distinctly higher average LC3II level later in the time course, suggesting a potentially higher available number of autophagosomes in the long term in the MSCs following a brief bafilomycin treatment at the onset of differentiation. These results suggest that standard osteogenic differentiation cues mobilize the accumulated autophagosomes in MSCs within 3 to 6 hours, which is followed by a ‘re-loading’ of autophagosomes following the utilization of the initial flux. Furthermore, the ability of this differentiation-stimulated flux to be inhibited by drug treatment suggests that this phenotype may be able to be exploited during differentiation, depending on clinical goals, and ultimately supports the hypothesis of a critical role for autophagosome accumulation and utilization in MSC differentiation.

**Figure 3:**
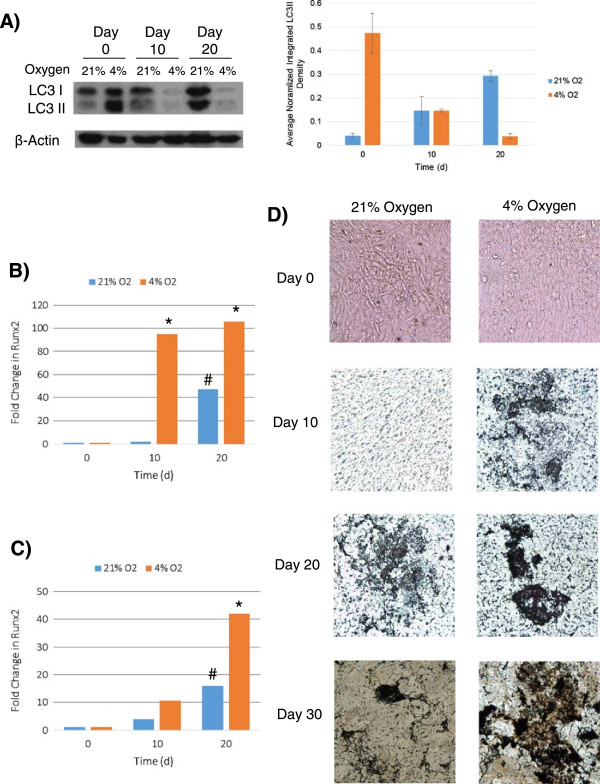
**Autophagy mobilized during differentiation of immortalized human mesenchymal stem cells (ihMSCs).** Cultured ihMSCs were stimulated to undergo osteogenesis to form osteoblasts for 30 days at 21% and 4% oxygen. LC3I and LC3II levels were assessed via immunoblot every 10 days and normalized to β-actin to derive average LC3II band densities **(A)**. Levels of Runx2 **(B)** and Osteocalcin **(C)**, markers of early and late osteogenesis, respectively, were assayed via quantitative polymerase chain reaction (^#^ *P* <0.05, *P <0.01). A Von Kossa stain was employed every 10 days to visualize bone spicule deposition at both oxygen concentrations **(D)**. The representative of experiments repeated at least three times is shown, and statistical significance is noted for difference from day 0 and between the two different ambient oxygen concentrations. Immunoblot normalized integrated densities were calculated and averaged by using a ratio of LC3II to beta-actin via sum of replicates for each blot. LC3, light chain 3.

Given the long-term nature of MSC differentiation and the need for energy generation during potential stressors such as hypoxic conditions, we also assayed how LC3 levels were affected during differentiation of MSCs into osteoblasts over 30 days in standard culture (21%) or more rapidly under the more physiologically relevant (4%) oxygen concentrations. Differentiation proceeded as expected, and hydroxyapatite deposition was seen by using Von Kossa stain after 10 days at 4% oxygen and after 20 days at 21% oxygen (Figure 3D). qPCR analysis of early and late osteogenesis markers (Runx2 and Osteocalcin levels) confirmed this, and more rapid differentiation was seen at 4% oxygen concentrations (Figure 3B and C). In terms of the autophagy phenotype, autophagosome marker LC3II was generally decreased by differentiation, relative LC3II levels fell by 10 days at 4% O_2_ and 20 days in 21% O_2_, and the different rates coincided with the bone deposition seen in culture (Figure 3A). LC3 expression on the whole also diminished during differentiation, as seen particularly in the more robust differentiation at 4% oxygen that resulted in reduced band intensity for both LC3I and LC3II, suggesting turnover of both LC3 pools with resultant decreases in autophagic potential as the cells form osteoblasts.

Differences in LC3 reduction seem to be directly related to oxygen concentration, which clearly has an effect on the rate of bone deposition, both early- and late-stage. Differentiation was much more rapid at 4% oxygen, which coincided with the more rapid LC3II reduction. This result suggests that MSCs may release from their unfused autophagosome state during times of high energy demand, such as during differentiation. Ultimately, the ‘pre-autophagy’ phenotype is seen only in functional and undifferentiated MSCs and may be a hallmark of such a cell.

### Modulation of differentiation outcomes through autophagy regulation

Data in Figures 2 and 3 suggest a key role for autophagy in MSC differentiation, particularly during the early critical period as the cells initiate the differentiation process. Earlier work demonstrated limited adipose tissue in mice lacking the critical effector *Atg7* (autophagy-related gene 7) in adipocytes [28, 29]. We hypothesized that the nature of autophagy’s apparent utility in differentiation would lead to altered differentiation outcomes if autophagy was modulated in the key early window of the process in general and thus examined a second differentiation pathway of adipogenesis. To test this, we differentiated immortalized MSCs into adipocytes by using commercial adipogenic media over the course of 30 days, in the face of 5 μM rapamycin or 5 nM bafilomycin treatments during the first 3 hours of differentiation (as in Figure 2) to alter the autophagic balance at the onset of differentiation.

**Figure 4:**
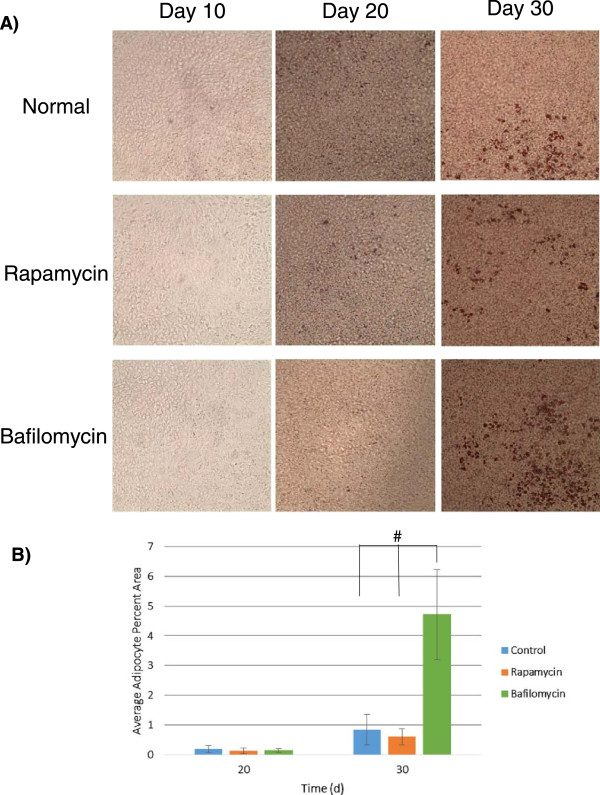
**Early arrested autophagy can be modulated to alter immortalized human mesenchymal stem cell adipogenesis outcomes. (A)** Immortalized human mesenchymal stem cells differentiated under adipogenic conditions were left untreated or treated with 5 μM rapamycin or 5 nM bafilomycin for the first 3 hours of differentiation. Differentiation outcomes were monitored via changes in fat droplet accumulation by using an Oil Red O stain. **(B)** Fat droplet percentage area in individual images was quantified by using thresholded images to monitor adipocyte formation. Analysis was performed by using representative images from eight-image fields across two studies (^#^ *P* <0.05).

Surprisingly, fat droplet accumulation via Oil Red O staining showed that bafilomycin treatment to block the autophagosome utilization early in differentiation led to a more robust differentiation/fat droplet accumulation over 30 days in the MSCs (Figure 4A). Quantification of fat droplets on day 20 showed little difference among treatments, and this was presumably due to the low amount of mature adipogenesis in general to this time point (Figure 4B). However, at day 30 when substantial fat accumulation was seen in culture, adipocyte percentage area quantification showed a significant increase in adipocyte accumulation in the bafilomycin-treated condition. This stands in contrast to the rapamycin treatment, which showed a slight decrement (that was not statistically significant) compared with the control differentiation. Thus, our results show that briefly delaying the induction of autophagy in adipogenic differentiation improves long-term differentiation outcomes and suggests a potential key role for autophagy and the balance of autophagosome accumulation/cycling in MSCs early in commitment to lineage. Additionally, we show here that autophagy plays a role across multiple differentiation pathways, suggesting a key role in general MSC physiology and potential clinical utility in many contexts.

### Autophagic turnover is halted in mesenchymal stem cells

MSCs analyzed by TEM showed a high concentration of autophagosome structures (Figure 1). However, single time points seen in TEM images did not reveal whether these led to degradation of the contents. The absence of late-stage autophagosomes hinted at an arrest in cycling, but nothing definitive could be garnered by these snapshots. Given the apparent dynamic role of autophagy in differentiation and the rapid change in phenotype seen in these cells, we examined the baseline status of any autophagic turnover in normal MSCs. To examine maturation and flux of autophagosomes over time, we used a tandem GFP-RFP LC3 plasmid to monitor the local pH environment of LC3II puncta. This plasmid tags LC3 in the cell with both GFP and RFP, and thus when LC3II is present in non-acidified autophagic structures, the green and red co-localize to show a yellow tag. However, the acidic environment necessary for lysosomal degradation of the autophagosome quenches the GFP signal, and thus any autophagosomes that have fused with active lysosomes will show only the RFP signal. This therefore represents a useful tool for monitoring autophagosome turnover in real time [21].

**Figure 5:**
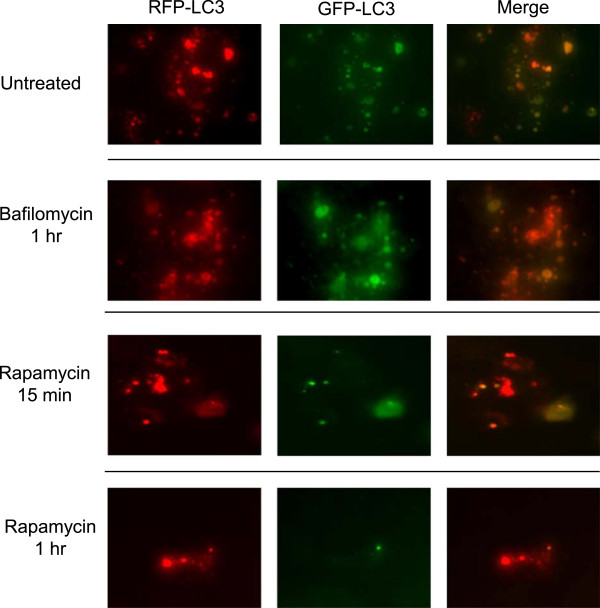
**Immortalized human mesenchymal stem cells show little autophagosome turnover unless stimulated.** Immortalized human mesenchymal stem cells grown under normal culture conditions were tagged with a tandem green fluorescent protein-red fluorescent protein (GFP-RFP) light chain 3 (LC3) via viral transfection (see Materials and methods). ihMSCs were then stimulated with bafilomycin to halt autophagy for 1 hour or with rapamycin to induce autophagy for 15 minutes and 1 hour. The representative of experiments repeated at least three times is shown.

Immortalized MSCs showed very little loss of GFP signal over time, indicating a lack of turnover of LC3II and suggesting arrested autophagic degradation in the cells (Figure 5). The yellow signal from GFP/RFP co-localization was quickly lost after 15 minutes of treatment with rapamycin to induce autophagy, and almost all GFP signal was quenched after 1 hour in rapamycin. Furthermore, treatment with bafilomycin to halt autophagy showed no appreciable effect on fluorescent signal in the ihMSCs. Given that bafilomycin halts autophagy by preventing lysosomal fusion and degradation, this phenotype would be expected in cells that already are arrested in maturation prior to forming acidified autolysosomes. Ultimately, little turnover of GFP signal (and thus autophagosome recycling) is seen unless stimulated with rapamycin. This suggests that, although MSCs do have a significant level of basal autophagosome formation, a great deal of these autophagosomes appear to be arrested in the middle of the autophagic process prior to lysosomal degradation. The cells are poised to be rapidly released from this state when cues signaling a need for autophagic degradation products are received (as occurs with mTOR inhibition).

## Discussion

Macroautophagy exists to quickly generate energy substrates during cellular stress due to nutrient deprivation, to remove unnecessary organelles during differentiation state changes, and/or to generate precursors for macromolecular syntheses. Understanding of its role and even its existence, in stem cell biology remains nascent, particularly in MSCs. Previous studies of autophagy in stem cells in general have shown a slightly nebulous but suggestive role of the autophagic processes in normal cell function and differentiation. Embryonic stem cells, for example, have been claimed to have constitutive levels of autophagy on the basis of an upregulation of autophagy proteins early during differentiation [30]. Additionally, reprogramming of somatic cells to induced pluripotent stem cells has been shown to involve the process of autophagy and its signaling pathways [31, 32]. Limited study of MSC autophagy has shown a significant constitutive level of autophagy-associated proteins to be upregulated in these cell, with potential early activation and, later, loss of the phenotype during differentiation [17, 18]. Additionally, production of the autophagic machinery has been shown to have a protective role during MSC starvation [16]. These reports all delineate a possible role of the process in generating energy or precursors for stem cells during times of stressed metabolic demand, such as differentiation, reprogramming, or starvation. Herein, we show that in an undifferentiated state, MSCs present an arrest in autophagy prior to autophagosome degradation by the lysosome. This effect is seen in both immortalized and primary cell lines, though to a less dramatic extent in the primary cells. Most importantly, the autophagic phenotype is lost during cellular differentiation.

**Figure 6:**
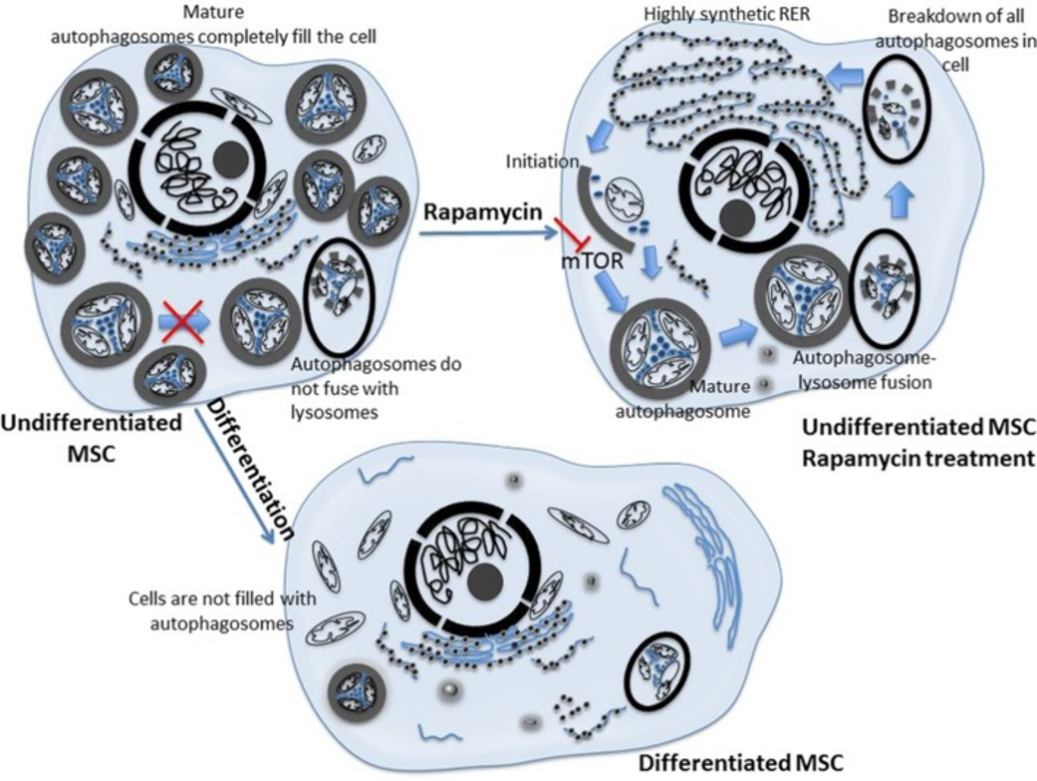
**Schematic for arrest and utilization of autophagy in normal mesenchymal stem cell (MSC) function.** As evidenced by transmission electron microscopy images and green fluorescent protein-red fluorescent protein tracking of light chain 3 in this study, undifferentiated MSCs appear to exist at a state of high basal autophagy, with many autophagosomes in a state of arrest in the cytosol (left). Artificial activation of autophagosome degradation with rapamycin (right) leads to a rapid clearance of existing autophagosomes in the cell, with subsequent rough endoplasmic reticulum (RER) enlargement, suggesting a potential advantage for MSCs to mobilize autophagy during times of high energy demand. Thus, during a time such as differentiation or nutrient starvation (bottom), a similar phenotype is observed in the cells whereby arrested autophagosomes in the cell are mobilized and degraded, allowing the MSCs to proceed in the differentiation process or potentially resist negative effects of stressors. mTOR, mammalian target of rapamycin.

Given the high concentration of autophagosomes in MSCs, the key role in differentiation, and the general lack of autophagic turnover at a basal state, we propose a model wherein MSCs exist in a state of ‘arrested’ autophagy, where an unusually high concentration of autophagosomes sit in the cells prior to lysosomal degradation (Figure 6). This arrested phenotype could be used to rapidly generate amino acid building blocks when necessary via autophagy, as indicated by RER enlargement following rapamycin stimulation in Figure 1. Although this RER enlargement is less pronounced in the primary cells, the mechanism is still present and could be less dramatic because of fewer autophagosomes present at baseline in primary cells. For example, cells undergoing differentiation may take advantage of this phenotype to rapidly generate precursors during the demanding differentiation process in a wound arena with limited perfusion or might take advantage of these arrested energy stores for situations where the cells are challenged to survive, such as in clinical cell therapy settings, including chronic skin wounds or myocardial defects.

A halted autophagic process may in fact be a hallmark of undifferentiated MSCs. Moreover, this novel autophagic characteristic of MSCs may play a key role in their physiology during wound healing. Cells in a wound environment are subject to a variety of stressors and challenges to their survival, making mechanisms like autophagy key for allowing stem cells to exist long enough to proliferate and differentiate effectively into the target cell type. In arresting autophagy before lysosomal degradation, MSCs have a method to rapidly release substrates for lysosomal degradation, generating energy and anabolic resources for the cell, as seen in the accompanying RER expansion. Additionally, although glycolysis is significant in MSCs [22, 33], it is possible that by resynthesizing mitochondria via this mechanism, the MSCs present the machinery for rapid macromolecule synthesis in nutrient-rich environments while providing materials for autophagic degradation and recycling of macromolecular building blocks. This mechanism may play a key role in how MSCs function in a wound bed and could also be manipulated to promote cell survival further in cell transplant scenarios, in which MSCs are subject to stressors that might activate an autophagic response in the cell.

Given the role of autophagy activation early in MSC differentiation that we have discussed here, our results showing the altered differentiation outcomes present an interesting possibility for affecting MSC differentiation across a variety of scenarios. Here, we showed inhibiting the autophagic flux activated by MSCs at the initiation of differentiation with a modest bafilomycin treatment helped the cells produce a more robust adipogenic differentiation over the course of 30 days. Notably, this result stands in apparent contrast to the mouse model that is deleted for *Atg7*, a key regulator of autophagy, specifically in adipocytes [29]. Studies using this model showed that the constitutive impairment of autophagy in the knockout mice significantly reduced adipogenesis, suggesting a key role for autophagy in the adipogenic process. At a high level, this appears to be discrepant with the results herein. However, the autophagy blockades in these two models are substantially different. Impaired autophagy in the *Atg7* knockout is on a permanent basis, disrupting autophagy and autophagosome cycling in fat beyond early stages of a differentiation process. Our results from a transient blockade of autophagy just during the earliest stages of differentiation support an activation of autophagy during early differentiation but appear to differ from the effects of the long-term autophagy blockade. For a transient inhibition, during a key early period in the initiation of adipogenesis where an innate accumulation of autophagosomes exists, preventing the cells from immediately cycling autophagosomes as they normally do may in fact allow the cells to more effectively use the autophagosome accumulation we have shown here. In total, these findings suggest that MSCs may ultimately benefit from delaying the onset of autophagic cycling to preserve the autophagosome balance beyond the normal time to autophagosome degradation.

By altering this autophagic phenotype early in the process and then allowing normal autophagy to occur, the cells are seemingly able to undergo a more efficient differentiation. Furthermore, we propose that holding the cells from cycling autophagosomes too early in the process may be beneficial to particular differentiation pathways and that placing cells in a pro-autophagy environment such as nutrient stress or hypoxia might hinder adipogenic differentiation if the MSCs are unable to maintain the autophagosome balance and activate cycling at the appropriate time. Additionally, this suggests that drugs to modulate autophagy might be tangentially used to affect desired differentiation outcomes in culture settings or also *in vivo* if a differentiation is desired in a clinical setting. Further study of this phenotype across various differentiation pathways is warranted, particularly establishing the specific pathway of activation that links differentiation and autophagy activation and also examining the mechanism of autophagosome accumulation that is the underlying key to this unique activity of MSCs. Although the phenotype as it relates to LC3 levels was qualitatively similar across both pathways studied here, more detailed analysis of specific utilization of autophagosomes and the utility of modulating autophagy is warranted in MSCs.

## Conclusions

Often hMSCs are placed into scenarios where metabolic demand is high and the environment demands increased energy output to function, such as cell stress in a cell transplant site or during differentiation. Autophagy is a well-known process by which MSCs might mobilize autophagic degradation to induce recycling of components for protein production and other key energy factors. Herein, we show that autophagosome concentrations are extremely high in MSCs at a basal state, showing an arrested phenotype prior to lysosomal degradation. This arrest is easily mobilized by stimulating conditions, leading to increased RER size and normal autophagy after mobilization. Furthermore, autophagy is mobilized very early in the process of differentiation in MSCs, confirming a key role for autophagy in the differentiation process. As such, altering the state of autophagy during the early stages of differentiation changes the long-term differentiation efficiency of the MSCs. Our results here further support a key role for autophagy in several differentiation pathways in MSCs and also present a potential utility for autophagy modulation in optimizing MSC differentiation or utility in cell therapy to improve therapeutic benefit.

## Abbreviations

atg7: autophagy-related gene 7
DMEM: Dulbecco’s modified Eagle’s medium
FBS: fetal bovine serum
GAPDH: glyceraldehyde 3-phosphate dehydrogenase
GFP: green fluorescent protein
hMSC: human mesenchymal stem cell
ihMSC: immortalized human mesenchymal stem cell
LC3: microtubule-associated proteins 1A/1B light chain 3A
MSC: mesenchymal stem cell/multipotent stromal cell
mTOR: mammalian target of rapamycin
PBS: phosphate-buffered saline
prhMSC: primary human bone marrow-derived multipotential stromal cell
qPCR: quantitative polymerase chain reaction
RER: rough endoplasmic reticulum
RFP: red fluorescent protein
TEM: transmission electron microscopy.
